# Cardiovascular Events and Management Strategies Including Tyrosine Kinase Inhibitor Switching, Dose Reduction, and Discontinuation in Chronic Myeloid Leukemia: A Single-Center Retrospective Study

**DOI:** 10.7759/cureus.105578

**Published:** 2026-03-21

**Authors:** Nobue Sato, Ryo Yoshimaru, Yong-Mei Guo, Hirotaka Nakamura, SungGi Chi, Kensuke Matsuda, Yosuke Minami, Junichiro Yuda

**Affiliations:** 1 Pharmaceutical, National Cancer Center Hospital East, Kashiwa, JPN; 2 Hematology and Oncology, National Cancer Center Hospital East, Kashiwa, JPN

**Keywords:** adverse cardiovascular events, bosutinib, chronic myelogenous leukemia (cml), dasatinib, imatinib, nilotinib, ponatinib, treatment-free remission, tyrosine kinase inhibitors (tki)

## Abstract

The development of tyrosine kinase inhibitors (TKIs) has dramatically improved the treatment outcomes of the chronic myelogenous leukemia-chronic phase (CML-CP). However, cardiovascular events (CVEs), such as hypertension, peripheral arterial occlusive disease, pulmonary arterial hypertension, and cerebral infarction, continue to occur, which highlights the need to develop preventive monitoring and treatment strategies for adverse vascular events. This study retrospectively analyzed the data on CVEs in 63 patients with chronic myeloid leukemia treated with TKIs at a single center between January 1, 2013, and October 31, 2023. TKI treatments were discontinued in one patient and 16 patients owing to clinically significant CVEs and adverse events other than CVEs, respectively. Male patients tended to experience more subclinical cardiovascular abnormalities with increasing age than female patients. No prolongation of the QT interval was observed. During follow-up, improvements in tricuspid regurgitant pressure gradient, brain natriuretic peptide values, and hypertension were observed after reducing the dose of the same TKI, interrupting the TKI treatment, and switching from the newer-generation TKIs to imatinib (IM) or bosutinib (BOS). These findings highlight the importance of blood pressure control and cardiovascular monitoring for CVE prevention in high-risk CML patients. Switching to IM or BOS, TKI dose reduction, or treatment discontinuation was observed to be associated with improvement in cardiovascular parameters in selected patients achieving deep molecular response.

## Introduction

Treatment outcomes of chronic myelogenous leukemia-chronic phase (CML-CP) have improved dramatically with the development of tyrosine kinase inhibitors (TKIs) [1-4]. In Japan, the following TKIs have been indicated for the treatment of CML: imatinib (IM), a first-generation TKI; nilotinib (NIL), dasatinib (DAS), and bosutinib (BOS), second-generation TKIs; and ponatinib (PON), a third-generation TKI with efficacy against the T315I mutation [4]. In the Ponatinib Ph+ ALL and CML Evaluation (PACE) trial of patients with relapsed and refractory Ph-positive leukemia treated with PON, the incidence of arterial occlusive events (AOEs) in these patients was 31% [4]. On the other hand, in the Optimizing Ponatinib Treatment in CP-CML (OPTIC) trial, in which the dose of PON was reduced from 45/30 mg to 15 mg after the *BCR-ABL* international scale (IS) ≤ 1% was achieved, the incidence of AOEs was reduced to 3.2% without compromising efficacy [5].

Treatment-free remission (TFR), defined as the maintenance of molecular remission after discontinuation of TKI therapy, has recently been regarded as a goal of TKI treatment in patients who achieve deep molecular response (DMR) over a sustained period. Factors contributing to TFR achievement include TKI treatment duration, periods from initiating TKI treatment to MMR achievement, and complete molecular response (CMR) duration [6,7]. The rate of patients who achieved DMR is higher in those treated with second-generation TKIs than in those treated with IM. Second-generation TKIs provide good effects in early treatment stages and are expected to increase the rate of patients who achieve TFR [7].

The onset of cardiovascular events (CVEs), such as AOEs [1,2,4,8], pulmonary arterial hypertension [9,10], and prolongation of the QT interval [11,12], has been reported in patients who receive PON, NIL, or DAS. The onset rates of CVEs are 5% and 2% in the DAS and IM groups (Dasatinib versus Imatinib Study in Treatment-Naïve CML Patients (DASISION) trial) [1], respectively, and 16.5%, 23.5%, and 3.6% in the NIL 600 mg/day, NIL 800 mg/day, and IM 400 mg/day groups (Evaluating Nilotinib Efficacy and Safety in Clinical Trials of Newly Diagnosed Patients (ENESTnd) trial) [2], respectively. The incidence of CVEs is higher in patients treated with second-generation TKIs and increases in a dose-dependent manner. We included 63 patients with CML-CP who received TKIs at our institution. We identified real-world data on CVE development and discussed CVE development, TKI changes, and dose adjustments. The primary objective of this study was to describe the spectrum of cardiovascular findings (subclinical abnormalities and clinically significant CVEs) and to evaluate the clinical course following TKI modification (dose reduction, switching, or discontinuation) in routine practice.

A previous version of this article was posted to the medRxiv preprint server on April 27, 2020.

## Materials and methods

Study design

This was a single-center, retrospective observational study conducted at the National Cancer Center Hospital East, Japan. The study aimed to evaluate the spectrum of cardiovascular findings, including subclinical cardiovascular abnormalities and clinically significant cardiovascular events, cardiovascular risk profiles, and the clinical course following TKI modification (dose reduction, switching, or discontinuation) in patients with CML-CP treated in routine clinical practice.

Patient recruitment

Patients with CML-CP who received at least one TKI between January 1, 2013, and October 31, 2023, were retrospectively identified using electronic medical records. Eligible patients were required to have at least one month of follow-up after TKI initiation. This minimum threshold was used to avoid excluding patients who discontinued treatment early because of intolerance or adverse events and to better reflect real-world clinical practice; however, it was not intended to indicate that one month is sufficient to evaluate molecular response or longer-term cardiovascular toxicity. No additional exclusion criteria were applied in order to reflect real-world clinical practice. All consecutive eligible patients treated during the study period were included.

Data collection

Clinical data were extracted from electronic medical records and included patient demographics (age and sex), TKI treatment history (type, dose, duration, switching, interruption, or discontinuation), comorbidities, and concomitant medications (anticoagulants, antiplatelet agents, lipid-lowering agents, antihypertensive agents, and hypoglycemic agents). Baseline cardiovascular comorbidities, including hypertension, diabetes mellitus, dyslipidemia, smoking history, chronic kidney disease, and prior cardiovascular disease, are summarized in Table 1.

**Table 1:** Definition of cardiovascular events This table defines the parameters and thresholds used to identify abnormal cardiovascular examination findings and mild cardiovascular events in this study. Abnormalities detected by ultrasonographic cardiography (UCG) and electrocardiogram (ECG) are listed, along with specific cutoff values for tricuspid regurgitant pressure gradient (TRPG ≥30 mmHg), QTc prolongation (≥500 ms, CTCAE Grade 3), elevated brain natriuretic peptide (BNP ≥100 pg/mL), hypertension (systolic blood pressure ≥140 mmHg and/or diastolic blood pressure ≥90 mmHg, CTCAE Grade 2), increased cardiothoracic ratio (CTR ≥50%), and pleural effusion on chest radiography. These definitions were established based on established cardiovascular and heart failure guidelines and the Common Terminology Criteria for Adverse Events (CTCAE) version 4.0 [13]. UCG, ultrasound cardiography; ECG, electrocardiogram; TRPG, transtricuspid pressure gradient; CTR, cardiothoracic ratio; BNP, plasma brain natriuretic peptide; sBP, systolic blood pressure; dBP, diastolic blood pressure; CTCAE, Common Terminology Criteria for Adverse Events

Examination	Parameter
		UCG	Mild left ventricular hypertrophy
Abnormalities	Left atrial enlargement
	Hemopericardium
	Left ventricular hypertrophy
	Cardiomegaly
	Old myocardial infarction
ECG abnormalities	QTc prolongation
	ST-segment elevation
	T-wave abnormality
	R-wave progression
	ST depression
	Left ventricular dilatation
	Left ventricular hypertrophy
	Nonspecific ST and T wave change
	Nonspecific ST-segment elevation
	Nonspecific T wave abnormalities
Increase of TRPG	≥ 30 mmHg
QTc prolongation	CTCAE (version 4.0): ≥ grade 3
	(QTc ≥ 500 ms)
High BNP levels	≥ 100 pg/mL
Hypertension	CTCAE (version 4.0): ≥ grade 2
	(sBP ≥ 140 mmHg/dBP ≥ 90 mmHg)
Increase in CTR	≥ 50%
X-ray	Pleural effusion

Cardiovascular-related data included blood pressure measurements, laboratory findings, electrocardiography (ECG), echocardiography (ultrasonographic cardiography, UCG), chest radiography, and clinical diagnoses of cardiovascular events. Tricuspid regurgitant pressure gradient (TRPG) and brain natriuretic peptide (BNP) levels were collected when available. Troponin testing was performed in patients with suspected myocardial injury or ischemic heart disease; however, systematic troponin measurements were not included because routine testing was not performed for all patients. ECG, BNP, UCG, and TRPG assessments were performed as part of routine clinical practice (at clinicians' discretion and according to clinical needs) rather than at predefined standardized intervals.

Outcome definitions

For interpretability, cardiovascular findings were categorized into (1) subclinical cardiovascular abnormalities and (2) clinically significant cardiovascular events requiring medical intervention. Subclinical cardiovascular abnormalities were defined as biomarker or imaging abnormalities detected through routine monitoring without a clinically diagnosed cardiovascular event, including elevated BNP (≥100 pg/mL), elevated TRPG (≥30 mmHg), abnormal ECG or UCG findings, Grade 2 or higher hypertension, increased cardiothoracic ratio (≥50%), or pleural effusion (PE). Clinically significant cardiovascular events were defined as conditions requiring medical intervention or resulting in TKI modification, including ischemic heart disease, cerebral infarction, and myocardial damage. Both tiers were included in this analysis because, in routine clinical practice, subclinical abnormalities frequently informed TKI modification decisions and therefore represent clinically relevant findings for cardiovascular monitoring. These definitions were based on established cardiovascular and heart failure guidelines [13].

Severe cardiovascular events were defined as ischemic heart disease, cerebral infarction, myocardial damage, or other cardiovascular conditions requiring medical intervention or resulting in TKI discontinuation. A TKI interruption was defined as discontinuation of TKI treatment for a period of at least one month. For patients who discontinued TKI therapy to attempt TFR, molecular relapse was defined as loss of MR3.0 requiring re-administration of TKI therapy.

Cardiovascular risk assessment tools

Baseline cardiovascular risk was assessed prior to initiation of any TKI using validated risk assessment tools, including the Suita Score [14], SCORE2 Risk Chart [15], and SCORE2-Older Persons (SCORE2-OP) Risk Chart [16]. These validated tools have been previously published as open-access resources: the Suita Score is freely available for clinical and research use in the original publication, and the SCORE2 and SCORE2-OP risk charts are published as open-access articles by the European Heart Journal under Creative Commons licensing. No additional permissions were required for their use in this study. Patients were categorized into low-, intermediate-, or high-risk groups according to published guideline thresholds for each scoring system.

Sample size consideration

Because this was an exploratory retrospective observational study with a descriptive design, a formal sample size calculation was not performed a priori. Instead, all consecutive eligible patients treated during the study period (January 1, 2013, to October 31, 2023) were included to maximize data completeness and to reflect real-world clinical practice. This consecutive sampling approach is consistent with methodological recommendations for retrospective cohort studies in which the primary aim is to describe clinical outcomes and generate hypotheses rather than to test a specific hypothesis; however, the resulting sample size may limit statistical power and generalizability.

Statistical analysis

Associations between CVE occurrence and cardiovascular risk scores (Suita Score, SCORE2 Risk Chart, and SCORE2-OP Risk Chart), as well as abnormal cardiovascular examination findings, were evaluated using Pearson's correlation coefficient. Statistical analyses were performed using EZR (Saitama Medical Center, Jichi Medical University, Shimotsuke City, Japan), a graphical user interface for R (R Development Core Team, Vienna, Austria). A p-value <0.05 was considered statistically significant [17].

Ethical considerations

This study was approved by the Institutional Review Board of the National Cancer Center, Japan, and was conducted in accordance with the Declaration of Helsinki. Patient consent was obtained through an opt-out informed consent process.

## Results

Patient characteristics

**Figure 1:**
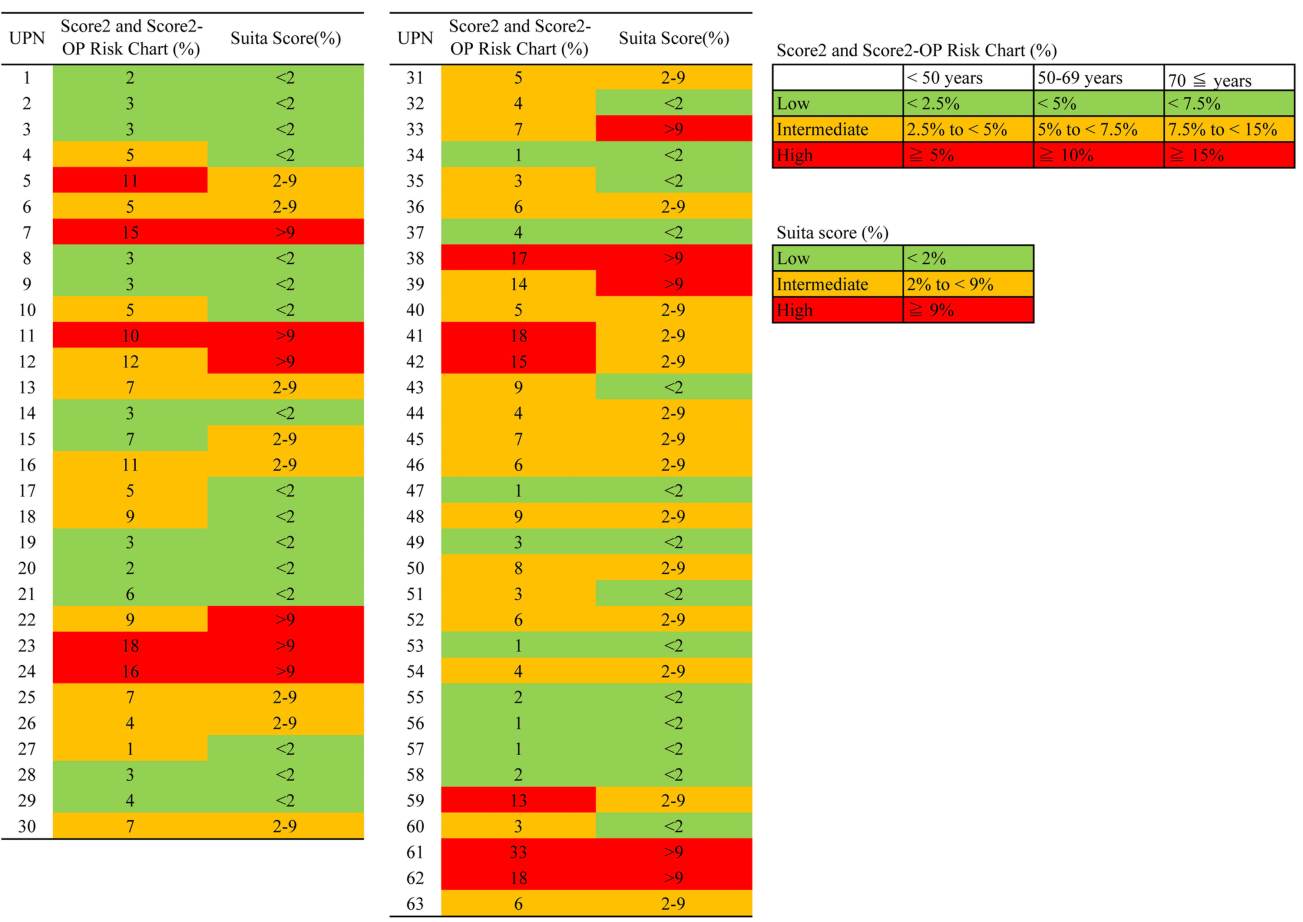
Estimated 10-year cardiovascular disease risk for the 63 patients Using the Suita score [14], SCORE2 [15], and SCORE2-OP [16] risk charts, cardiovascular disease risk was evaluated, and 63 patients were classified into three risk categories: low (green), intermediate (yellow), and high (red). The unique patient number (UPN) corresponds to the 63 CML patients included in this study. Risk categories were defined according to published guideline thresholds for each scoring system.

The data on 63 patients are shown in Figure 1 and Table 2. We included 63 patients with CML-CP treated with TKIs (39 men and 24 women). The median ages at the initial examination, follow-up, and treatment periods were 56 (range: 19-86) years, 72 (range: 6-288) months, and 72 (range: 6-288) months, respectively. IM, NIL, DAS, and BOS were used as first-line treatment in 33 (52%), 15 (24%), 12 (19%), and 3 (5%) patients, respectively. The proportion of second-generation TKIs was higher than that of first- and third-generation TKIs in second-line treatment. Specifically, IM, NIL, DAS, BOS, and PON were used in 4 (11%), 13 (36%), 11 (31%), 7 (19%), and 1 patient, respectively. Regarding TKIs used as a third-line treatment, IM, NIL, DAS, BOS, and PON were used in six (40%), three (20%), two (13%), three (20%), and one patient, respectively. NIL, DAS, and BOS were administered as a fourth-line treatment to three (42%), two (29%), and two patients (29%), respectively (Table 3).

**Table 2:** Details of the cardiovascular profile of the 63 patients A hyphen (-) indicates that data are not available or not applicable. The following symbols were used to represent the patient status: ◆, patients who required TKI switching because of CVE onset; ▼, patients with reduced TKI dose because of CVEs; ●, patients who experienced CVE that required intervention but continued the same TKI at the same dosage; ○, patients who experienced a CVE that did not require intervention and continued the same TKI at the same dosage; and ■, patients in whom the TKI treatment was discontinued after CVE onset. UPN, unique patient number; TKIs, tyrosine kinase inhibitor; BNP, plasma brain natriuretic peptide; PE, pleural effusion; TRPG, transtricuspid pressure gradient; UCG, ultrasound cardiography; CTR, cardiothoracic ratio; ECG, electrocardiogram; HT, hypertension; CVE, cardiovascular event; DM, diabetes mellitus; CKD, chronic kidney disease; HL, hyperlipidemic

UPN	Underlying		CVE risk factor*	Evaluating CVE risk		CVE detail									
	Disease	Treatment		Score2 and Score2-OP Risk Chart (%)	Suita Score (%)	Discontinuing TKI	QTc prolongation	High BNP levels	PE	Increased TRPG	UCG abnormalities	Increased CTR	ECG abnormalities	HT	Other
1	-	Hypoglycemic drug	DM	2	<2	Yes	-	-	◆	○	■	○	■	-	Myocardiopathy■
2	-	-	-	3	<2	Yes	-	-	-	-	-	-	○	○	-
3	-	-	-	3	<2	Yes	-	-	-	-	-	-	-	-	-
4	-	-	CKD	5	<2	Yes	-	○	-	-	○	-	-	●	-
5	Chemical ionization	Antiplatelet drug	Smoker	11	2-9	Yes	-	-	-	-	-	○	○	●	Angina pectoris◆, Ischaemic heart disease◆
6	-	-	-	5	2-9	Yes	-	-	◆	○	-	-	-	●	-
7	Chemical ionization	Antiplatelet drug, antihypertensive drug, lipid-lowering drug, hypoglycemic drug	HL, HT, DM	15	>9	Yes	-	○	○	○	○	-	-	○	-
8	ECG abnormalities, HT	-	Smoker, HT	3	<2	Yes	-	-	-	-	-	-	○	○	-
9	-	-	Smoker	3	<2	Yes	-	-	-	-	-	-	○	-	-
10	-	-	Smoker	5	<2	Yes	-	-	-	-	-	-	○	-	-
11	-	Antihypertensive drug, lipid-lowering drug, hypoglycemic drug, antiplatelet drug	Smoker, HT, HL, DM	10	>9	Yes	-	-	-	-	-	-	-	○	-
12	Increased CTR	-	Smoker, CKD	12	>9	Yes	-	○	-	○	-	○	-	●	-
13	-	-	HT	7	2-9	Yes	-	-	-	-	-	-	○	○	-
14	-	-	Smoker	3	<2	Yes	-	-	○	-	-	-	○	-	-
15	Chemical ionization	Antihypertensive drug, lipid-lowering drug, antiplatelet drug	HT, HL	7	2-9	Yes	-	-	-	-	-	-	-	○	
16	ECG abnormalities	-	-	11	2-9	Yes	-	○	-	-	-	-	○	●	-
17	-	-	Smoker	5	<2	Yes	-	-	◆	-	○	○	○	○	-
18	Aortic stenosis	Anticoagulant drug, lipid-lowering drug, antihypertensive drug	-	9	<2	Yes	-	○	-	○	-	-	-	○	-
19	-	-	Smoker, HT	3	<2	Yes	-	-	-	-	-	-	-	○	-
20	-	-	-	2	<2	Yes	-	-	-	-	○	-	○	-	-
21	HT	Antihypertensive drug	HT	6	<2	Yes	-	-	-	-	-	-	-	-	-
22	ECG abnormalities	-	Smoker	9	>9	Yes	-	-	▼	-	-	-	○	-	-
23	ECG abnormalities, increased CTR	-	Smoker, HT, HL, CKD	18	>9	Yes	-	-	-	-	○	○	○	○	Angina pectoris◆
24	Bradycardia-tachycardia syndrome, HT	Anticoagulant drug, antihypertensive drug	Smoker, HT, CKD	16	>9	Yes	-	-	▼/◆	-	-	○	○	-	-
25	ECG abnormalities	-	Smoker, CKD	7	2-9	No	-	-	-	-	-	○	○	●	-
26	-	Antihypertensive drug	Smoker, HT	4	2-9	No	-	-	-	○	-	-	○	●	Angina pectoris▼
27	-	-	Smoker	1	<2	No	-	-	-	-	-	-	-	○	-
28	-	-	Smoker	3	<2	No	-	-	◆	-	-	-	○	○	-
29	-	-	Smoker	4	<2	No	-	-	-	-	○	○	○	●	-
30	-	-	Smoker, CKD	7	2-9	No	-	-	-	-	-	-	-	○	Arrhythmia● , Paroxysmal atrial fibrillation●
31	-	Hypoglycemic drug, antihypertensive drug	Smoker, HT, HL, DM	5	2-9	No	-	-	-	-	○	-	○	○	Cerebral infarction◆
32	-	-	Smoker	4	<2	No	-	-	-	-	○	-	-	○	-
33	-	-	Smoker	7	>9	No	-	-	-	-	-	○	○	○	-
34	-	-	-	1	<2	No	-	-	-	-	-	-	-	-	-
35	-	-	Smoker	3	<2	No	-	-	-	-	-	-	○	-	-
36	-	-	Smoker	6	2-9	No	-	-	-	-	○	-	○	-	-
37	-	Antihypertensive drug	HT	4	<2	No	-	-	-	-	○	○	○	-	-
38	ECG abnormalities	Hypoglycemic drug	Smoker, CKD	17	>9	No	-	-	-	-	-	○	○	●	-
39	Chemical ionization	Antiplatelet drug, antihypertensive drug	Smoker	14	>9	No	-	-	-	-	○	○	○	○	Myocardial infarction●
40	-	-	HL	5	2-9	No	-	-	-	○	-	-	-	-	-
41	-	Antihypertensive drug	Smoker, HT, CKD	18	2-9	No	-	○	◆	○	○	○	○	●	Angina pectoris●
42	ECG abnormalities	-	Smoker, CKD	15	2-9	No	-	-	-	○	-	○	○	-	Cerebral infarction●
43	MI, increased CTR	Antiplatelet drug, Antihypertensive drug	Smoker, HT, CKD	9	<2	No	-	○	-	-	-	○	-	-	-
44	-	-	Smoker	4	2-9	No	-	-	-	-	-	-	-	○	-
45	-	Antihypertensive drug	Smoker, HT	7	2-9	No	-	-	-	-	-	○	-	○	-
46	-	-	Smoker	6	2-9	No	-	-	-	-	-	-	-	-	-
47	Increased CTR	-	-	1	<2	No	-	-	-	-	-	○	-	-	-
48	ECG abnormalities, HT, high BNP levels	-	Smoker, HT	9	2-9	Yes	-	○	▼	-	○	○	○	●	-
49	-	-	Smoker	3	<2	No	-	-	-	○	-	-	-	-	-
50	-	-	HT	8	2-9	No	-	-	-	-	-	-	-	-	-
51	-	-	Smoker	3	<2	No	-	-	-	-	-	○	-	-	-
52	-	-	Smoker	6	2-9	No	-	-	-	-	-	-	-	-	-
53	-	-	-	1	<2	No	-	-	-	-	-	-	-	-	-
54	-	Lipid-lowering drug	Smoker, HL	4	2-9	No	-	-	-	-	○	-	-	-	-
55	-	Lipid-lowering drug, Antihypertensive drug	HL, HT	2	<2	No	-	-	-	-	-	-	-	-	-
56	-	-	-	1	<2	No	-	-	-	-	-	-	-	-	-
57	-	-	-	1	<2	No	-	-	-	-	-	-	-	-	-
58	-	-	-	2	<2	No	-	-	-	-	-	-	-	○	-
59	Arrhythmia, UCG abnormalities	Antihypertensive drug	Smoker, HT	13	2-9	No	-	-	-	-	-	-	○	-	-
60	-	-	Smoker, HL	3	<2	No	-	-	-	-	-	-	-	-	-
61	ECG abnormalities, HT	Antihypertensive drug	-	33	>9	No	-	-	-	-	-	-	○	-	-
62	HT	Antihypertensive drug	Smoker, HT	18	>9	No	-	-	-	-	-	-	-	○	-
63	UCG abnormalities	-	Smoker	6	2-9	No	-	-	-	-	-	-	-	-	-

**Table 3:** Types of TKI in each treatment line This table summarizes the distribution of tyrosine kinase inhibitors (TKIs) administered at each treatment line among the 63 patients with chronic myeloid leukemia in chronic phase (CML-CP). For each TKI, the number of patients and the median duration of treatment in months (with range) are presented. First-line treatments represent initial therapy choices at diagnosis, while subsequent treatment lines indicate TKIs used after switching due to intolerance, treatment failure, or adverse events. The decreasing sample sizes in later treatment lines reflect that not all patients required multiple TKI switches. TKI, tyrosine kinase inhibitor

Treatment line	Number of patients	Median duration of TKI treatment
		(months, range)
1st-line TKI (n=63)		
Imatinib	33	95 (1-227)
Nilotinib	15	24 (1-96)
Dasatinib	12	11 (1-80)
Bosutinib	3	3 (1-4)
2nd-line TKI (n=36)		
Imatinib	4	24 (4-28)
Nilotinib	13	41 (1-113)
Dasatinib	11	20 (1-135)
Bosutinib	7	24 (1-31)
Ponatinib	1	7.5
3rd-line TKI (n=15)		
Imatinib	6	7 (1-40)
Nilotinib	3	1 (1-28)
Dasatinib	2	3 (2-5)
Bosutinib	3	11 (2-13)
Ponatinib	1	2
4th-line TKI (n=7)		
Nilotinib	3	7 (1-100)
Dasatinib	2	6 (1-12)
Bosutinib	2	13 (1-25)
5th-line TKI (n=3)		
Bosutinib	1	2
Ponatinib	2	20 (1-40)
6th-line TKI (n=2)		
Nilotinib	1	43
Ponatinib	1	5

We investigated comorbidity, concomitant drugs (anticoagulant drugs, antiplatelet agents, lipid-lowering drugs, antihypertensive drugs, and hypoglycemic drugs); CVE risk evaluation based on SCORE2 Risk Chart, SCORE2-OP Risk Chart, or Suita Score (before starting any TKI); and the presence or absence of CVE onset (Figure 1). Fifty-two of the 63 patients developed CVE. Of these, nine patients required TKI switching because of CVE onset, four patients required reduced TKI dose because of CVEs, 14 patients who experienced CVEs and required intervention continued the same TKI at the same dosage, and one patient discontinued TKI treatment after CVE onset. Some patients had overlapping categories. Of 30 patients with CVE risk factors, such as cardiovascular diseases (cerebral infarction, hypertension, aortic stenosis, bradycardia-tachycardia syndrome, and myocardial infarction (MI)), type 2 diabetes, or dyslipidemia, 17 received interventions for lifestyle-related diseases (Table 2).

Cardiovascular events associated with TKI treatment

Before the initial TKI treatment, 22 patients had subclinical cardiovascular abnormalities. During the TKI treatment, subclinical cardiovascular abnormalities were observed. Specifically, Grade 2 hypertension (systolic blood pressure (sBP): ≥140 mmHg, diastolic blood pressure (dBP): ≥90 mmHg), elevated BNP, increased CTR on plain chest X-ray (≥50%), abnormal findings on ECG, elevated TRPG, and abnormal findings on UCG occurred in 32 (51%), 8 (13%), 19 (30%), 30 (48%), 10 (16%), and 15 (24%) patients, respectively; however, TKIs were continued at the same dose in all the patients. PE occurred in 10 (16%) patients. Owing to this adverse event, TKIs were switched in six patients. In the remaining four patients, TKIs were not switched: the dose was reduced in two (UPN: 22 and 48), and no intervention was performed in two (UPN: 7 and 14), who continued the same TKI at the same dose. Abnormal findings on UCG occurred in 15 patients, and one of them (UPN: 1) required TKI discontinuation because of depressed left/right ventricular systolic function, right heart strain, pericardial effusion, and PE. Abnormal ECG findings occurred in 30 patients, and one of them (UPN: 1) required TKI discontinuation because of T-wave inversion attributed to myocardial damage. Hypertension classified as Grade 2 (i.e., sBP ≥ 140 mmHg; dBP ≥90 mmHg) occurred in 32 of 63 patients (51%) after TKI initiation or switching. Of these, 11 patients (17%) required hypotensive therapy (Table 2). Nine patients experienced ischemic heart disease (angina pectoris (AP) and MI) or cerebral infarction. Specifically, two patients (UPN: 1 and 41) had PE, four (UPN: 1, 26, 41, and 42) had elevated TRPG, five showed abnormal findings on UCG, five had an increased CTR, and eight showed abnormal ECG findings (Table 2).

Prolongation of the QT interval (≥500 ms), an adverse event associated with NIL, did not occur (Table 4). Non-cardiogenic PE developed in 10 of 63 patients (16%).

**Table 4:** Results of the cardiovascular examination This table presents the incidence and clinical characteristics of cardiovascular abnormalities observed during TKI treatment in the study population. For each cardiovascular parameter, the following data are shown: total number of affected patients with percentage, median age at the time of CVE detection (years, with range), and median duration from TKI initiation to CVE onset (months, with range). The right columns display the distribution of affected patients across cardiovascular risk categories (low, intermediate, and high risk) as determined by the SCORE2 and SCORE2-OP Risk Charts prior to TKI initiation. No cases of QTc prolongation ≥500 ms were observed in this cohort. TKI, tyrosine kinase inhibitor; CVE, cardiovascular event; TRPG, transtricuspid pressure gradient; UCG, ultrasound cardiography; CTR, cardiothoracic ratio; BNP, plasma brain natriuretic peptide; ECG, electrocardiogram

Parameters	Total number of patients (%)	Age at incidence of CVE (range)	Duration of onset of CVE from initiating TKI (range)	Risk of CVE		
				Low (%)	Intermediate (%)	High (%)
Hypertension	32 (51)	70 (27-84)	85 (1-263)	3 (5)	14 (22)	14 (22)
BNP (> 100 pg/mL)	8 (13)	75 (62-84)	35 (2-165)	0	5 (8)	3 (5)
CTR (> 50%)	19 (30)	67 (30-83)	57 (1-184)	2 (3)	5 (8)	12 (19)
Pleural effusion	10 (16)	67 (34-83)	15 (1-107)	2 (3)	4 (6)	3 (5)
QTc prolongation (> 500 ms)	0	0	0	0	0	0
ECG abnormalities	30 (48)	67 (29-80)	66 (1-262)	4 (6)	11 (17)	12 (19)
TRPG (> 30 mmHg)	10 (16)	74 (34-84)	63 (8-185)	2 (3)	4 (6)	4 (6)
UCG abnormalities	15 (24)	67 (34-83)	67 (1-172)	2 (3)	7 (11)	6 (10)

Risk factors for cardiovascular events

There was little correlation between CVE occurrence (leading to treatment change or dose reduction) and TKI ≥3 lines (Pearson's correlation coefficient φ=0.01, p=0.92). Among patients with TKI ≥3, 6 of 17 had treatment changes due to non-CVE adverse events (skin rash, fever, colitis, gastrointestinal symptoms, herpes-zoster), which may have prevented the continuation of TKI until CVE onset. CVE development was associated with moderate or higher scores on the SCORE2 Risk Chart or SCORE2-OP Risk Chart (Pearson's correlation coefficient φ=0.26, p=0.04) and tended to be associated with moderate or higher scores on the Suita score (φ=0.18, p=0.15). Patients with moderate or higher scores on the SCORE2 Risk Chart or SCORE2-OP Risk Chart tended to be correlated with hypertension (Pearson's correlation coefficient φ=0.25, p=0.05) and significantly correlated with elevated BNP (φ=0.27, p=0.03).

Correlation of sex and age with CVE risk

The median age at the initial examination was compared among the following three groups: patients with subclinical cardiovascular abnormalities (subclinical cardiovascular abnormalities group), those with clinically significant cardiovascular events (clinically significant cardiovascular events group), and those without clinically significant cardiovascular events (no cardiovascular events group). The median age was 58 (range: 19-85), 66 (range: 27-78), and 38 (range: 28-70) years for men in subclinical cardiovascular abnormalities group, clinically significant cardiovascular events group, and no cardiovascular events group, respectively, and 58 (range: 40-75), 63, and 64 (range: 32-72) years for women in subclinical cardiovascular abnormalities group, clinically significant cardiovascular events group, and no cardiovascular events group, respectively. Male patients tended to experience more subclinical cardiovascular abnormalities with increasing age (Table 5), whereas female patients did not show such a tendency. Abnormal findings on cardiovascular examination were frequently observed in CML patients under TKI treatment; however, blood pressure control and periodical cardiovascular examination allowed the prevention or early detection of CVEs, which enabled the safe continuation of TKIs.

**Table 5:** Severity of cardiovascular events by patient background This table compares patient characteristics stratified by sex and cardiovascular event (CVE) severity. Patients were categorized into three groups: subclinical cardiovascular abnormalities group (patients with biomarker or imaging abnormalities detected through routine monitoring without a clinically diagnosed cardiovascular event, including ECG and UCG abnormalities), clinically significant cardiovascular events group (patients with cardiovascular events, such as ischemic heart disease, cerebral infarction, or myocardial damage requiring medical intervention or TKI modification), and no cardiovascular events group (patients without cardiovascular events). Median age at initial examination (years) and median duration of TKI treatment (months) are presented with ranges in parentheses. Male patients tended to experience more cardiovascular abnormalities with increasing age, whereas this trend was not observed in female patients. CVE, cardiovascular event; ECG, electrocardiogram; UCG, ultrasound cardiography; TKI, tyrosine kinase inhibitor *The subclinical cardiovascular abnormalities group includes patients with abnormal ECG and/or UCG findings that did not require TKI discontinuation or switching.

Parameters	CVE		Non-CVE	Total
	Mild* (n=42)	Severe (n=9)		
Age (years)				
Male	58 (19-85)	66 (27-78)	38 (28-70)	55 (19-86)
Female	58 (40-75)	63	64 (32-72)	62 (32-75)
Duration of treatment (months)				
Male	96 (2-279)	150 (6-254)	19 (2-144)	64 (2-279)
Female	78 (36-301)	208	17 (8-222)	64 (2-279)

SCORE2 Risk Chart and SCORE2-OP Risk Chart

The risk assessment of CVE onset based on the SCORE2 risk chart and SCORE2-OP Risk Chart showed that 21, 31, and 11 patients were classified into low-, moderate-, and high-risk groups, respectively (Figure 1, Table 2). The risk assessment of CVE onset based on the Suita Score indicated that 30, 22, and 11 patients were classified into low-, moderate-, and high-risk groups, respectively. Of the nine patients who experienced clinically significant cardiovascular events (AP, cerebral infarction, and MI) that required intervention, eight (89%) patients were categorized as a moderate- or high-risk group (details in Table 2) and the other patient (UPN: 1) was categorized to the low-risk group based on the SCORE2 Risk Chart, SCORE2-OP Risk Chart, and Suita Score.

Detailed clinical course of 63 patients under TKI treatment

**Figure 2:**
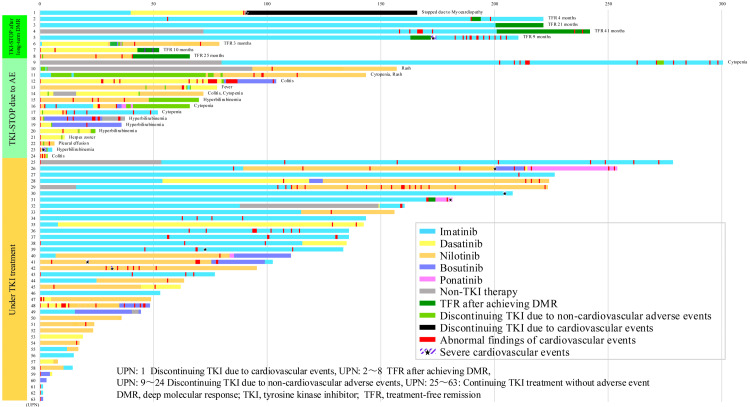
Detailed clinical course of the 63 patients under TKI treatment The bar graph shows treatment history with TKIs and the onset rate of CVEs in each patient with CML-CP. The X-axis is the observation period (month), and the Y-axis is the unique patient number (UPN). The clinical course of the following patients is shown: one patient who discontinued TKI because of severe CVE (UPN: 1), seven stopped TKIs after achieving long-term DMR (UPN: 2-8), 16 who discontinued TKIs because of adverse events other than CVEs (UPN: 9-24), and 39 who continued TKIs (UPN: 25-63). Imatinib treatment duration is shown in sky blue; dasatinib treatment duration, yellow; nilotinib treatment duration, orange; bosutinib treatment duration, medium slate blue; ponatinib treatment duration, magenta; treatment duration other than TKIs, gray; TFR duration referring to DMR, green; duration of TKI discontinuation because of adverse events other than CVEs, yellow-green; duration of TKI discontinuation because of CVEs, black; duration of abnormal finding occurrence on cardiovascular examination and of subclinical cardiovascular abnormality onset, red; and duration of severe CVE onset in violet stripe, triangle. UPN: 1 maintained treatment-free remission (TFR) for 75 months after TKI discontinuation because of myocardial damage. UPN: 2-8 discontinued TKIs because they maintained deep molecular response (DMR) for a long duration; however, TKIs were reinitiated in UPN: 2, 5, and 6 because they lost MR 3.0. UPN: 2-5 had an imatinib treatment history of ≥10 years, UPN: 6 and 7 had a dasatinib treatment history of ≥2 years. UPN: 8 had a nilotinib treatment history of ≥2 years. The following diseases were observed: hyperbilirubinemia (details in Table 2), cytopenia (details in Table 2), rash (UPN:10), colitis (UPN:24), gastrointestinal symptoms (UPN: 12,14), fever (UPN: 13), and herpes zoster (UPN: 21).

Figure 2 shows the presence or absence of TKI treatment and CVE status over time. TKIs were discontinued in one patient (UPN: 1) because of a clinically significant cardiovascular event. Other patients (UPN: 2-8) had their treatment discontinued at the patient's request, after achieving a deep molecular response. The TKIs were discontinued in one patient (UPN: 1) owing to clinically significant cardiovascular events and in 16 patients (UPN: 9-24) owing to adverse events other than CVEs. Additionally, TKIs were interrupted in seven patients after achieving DMR.

Discontinuation of TKI treatment

Among the seven patients (i.e., (UPN: 2-8) who maintained long-term DMR and in whom TKIs were discontinued to achieve TFR), four (UPN: 2-5) had an IM treatment history of ≥10 years, two (UPN: 6 and 7) had a DAS treatment history of ≥2 years, and one (UPN: 8) had a NIL treatment history of ≥2 years. Of the eight (UPN: 1-8) patients, three (UPN: 2, 5, and 6) patients lost MR 3.0, and the same TKI (i.e., the same TKI was used in these patients before and after the discontinuation) was readministered. DMR was maintained in the other five patients, and no TKI was administered. TKIs were discontinued in a total of 16 patients because of adverse events other than CVEs. Specifically, hyperbilirubinemia, cytopenia, and other adverse events (e.g., rash (UPN: 10), colitis (UPN: 24), gastrointestinal symptoms (UPN: 12 and 14), fever (UPN: 13), and herpes zoster (UPN: 21)) occurred in five (details in Table 2), five (details in Table 2), and six patients, respectively.

Changes in the CVE index (hypertension, TRPG, BNP) during TKI treatment

**Figure 3:**
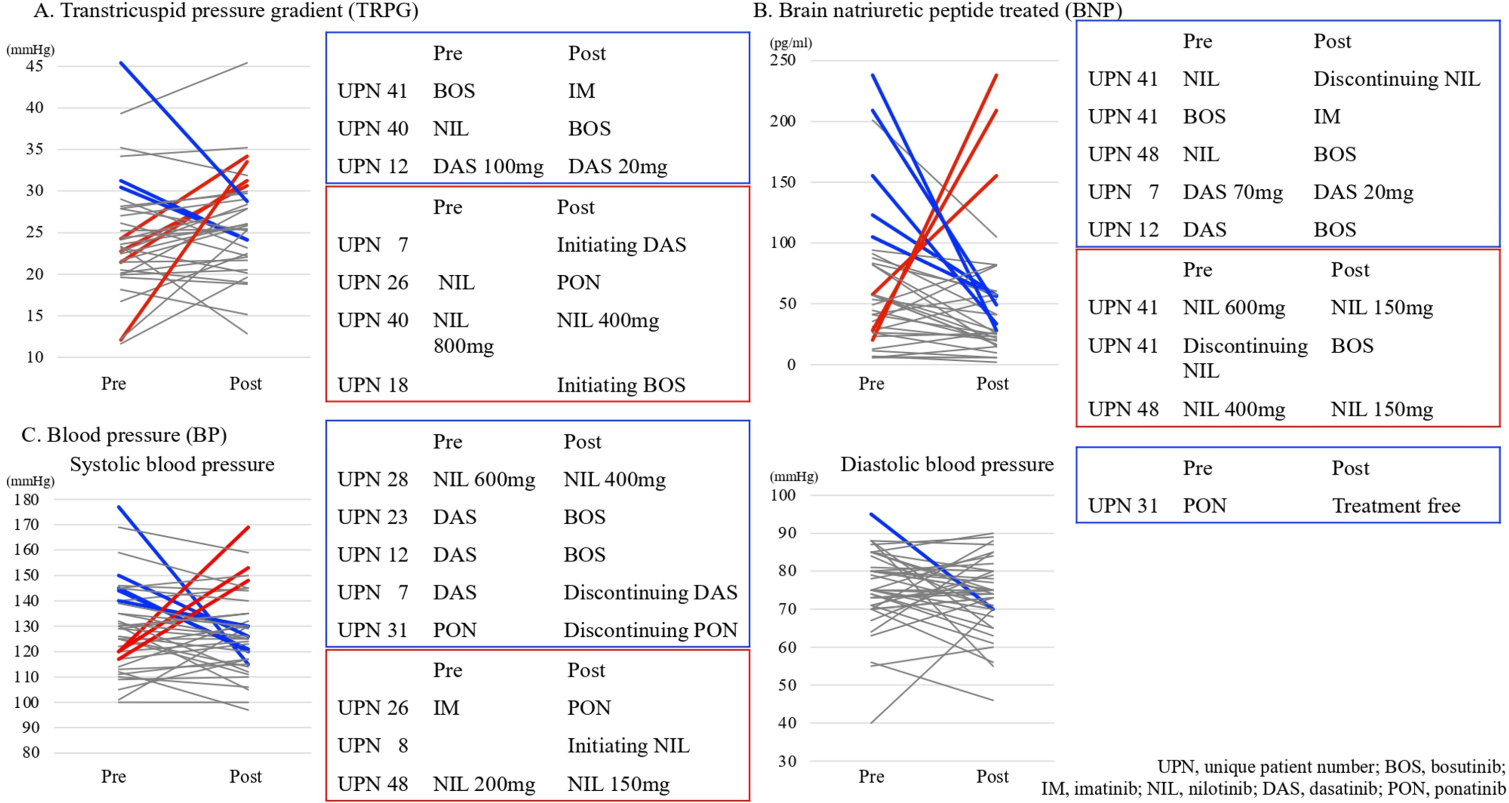
Changes in the indicators for cardiotoxicity during TKI treatment The figure illustrates changes in tricuspid regurgitant pressure gradient (TRPG; reference value <30 mmHg), brain natriuretic peptide (BNP; reference value <100 pg/mL), and systolic and diastolic blood pressure before and after tyrosine kinase inhibitor (TKI) switch/reduction and TKI discontinuation. Red lines indicate worsening, defined as a transition from normal to abnormal values (TRPG ≥30 mmHg, BNP ≥100 pg/mL, or development of hypertension defined as systolic blood pressure ≥140 mmHg and/or diastolic blood pressure ≥90 mmHg). Blue lines indicate improvement, defined as a transition from abnormal to normal values. “Pre” refers to measurements obtained immediately before the TKI modification, and “Post” refers to measurements obtained after switching, dose reduction, or discontinuation. A) The transtricuspid pressure gradient: TRPG data on 19 patients are available, and changes in their TRPG levels are shown. Elevated TRPG returned to normal in UPN: 41, 40, and 12. TRPG increased from the normal to high levels in UPN: 7, 26, 40, and 18. B) Brain natriuretic peptide (BNP): BNP data on 19 patients are available, and changes in their BNP levels are shown. Elevated BNP returned to normal in UPN: 41, 48, 7, and 12. BNP increased from the normal to high levels in UPN: 41 (on two separate occasions during treatment modifications) and 48. C) Blood pressure: Blood pressure data before and after TKI switching/discontinuation are available in 27 patients, and their systolic and diastolic blood pressures are shown. Grade 2 hypertension (systolic blood pressure: ≥140 mmHg, diastolic blood pressure: ≥90 mmHg) developed in UPN: 26, 8, and 48. Among patients with Grade 2 or severe hypertension, their blood pressure returned to normal in UPN: 28, 12, 23, 7, and 31.

Figure 3 shows the data on TRPG (reference value ≤30 mmHg), BNP (reference value <100 pg/mL) status, and sBP/dBP changes before and after TKI (switching/reduction/discontinuation) in 63 patients. The TRPG data of 19 patients were also provided. Of these, elevated TRPG returned to normal in the following three (16%) patients: BOS was switched to IM as a third-line treatment in one patient due to PE (UPN: 41); NIL was changed to BOS as a fourth-line treatment (UPN: 40) and DAS as a first-line treatment was reduced (UPN: 12). TRPG was increased from the reference to high values in the following four patients (21%): DAS as a first-line treatment (UPN: 7); NIL switched to PON as a fourth-line treatment (UPN: 26); NIL reduced as the second-line treatment (UPN: 40); and BOS as a second-line treatment after NIL (UPN: 18) (Figure 3A).

Data on BNP before and after TKI discontinuation were available for 19 patients with elevated BNP (≥100 pg/mL), which returned to normal in five (26%) patients; TKI was discontinued after completing the first-line treatment using NIL in one patient (UPN: 41); BOS was switched to IM as a third-line treatment in one patient (UPN: 41); NIL was switched to BOS as the third-line treatment in one patient (UPN: 48); DAS as a first-line treatment was reduced in one patient (UPN: 7); DAS was changed to BOS as a second-line treatment in one patient (UPN: 12); and BNP increased from the reference value to ≥100 pg/mL in three patients (16%). Specifically, NIL was reduced in two patients (11%) (UPN: 41 and 48). NIL was discontinued because of PE, and BOS was initiated as a second-line treatment in one patient (UPN: 41) (Figure 3B).

Elevated TRPG and BNP levels were observed after switching from the newer-generation TKIs to BOS or after IM and TKI discontinuation; however, such abnormal findings improved. Nevertheless, BNP increased after TKI reduction in two patients (UPN: 41 and 48) who had received NIL, a second-generation TKI, for >2 years. Before and after TKI switching, data on blood pressure were available in 27 patients. Of these, Grade 2 hypertension (sBP: ≥140 mmHg; dBP: ≥90 mmHg) developed in the following three patients (11%): UPN 26, who received PON as a fourth-line treatment after receiving IM as a first-line treatment; UPN 8, who initiated NIL as a first-line treatment; and UPN 48, in whom NIL was reduced (Figure 3C).

Grade 2 or higher hypertension became normal in the following five patients (18%): UPN 28, in whom NIL used as a fourth-line treatment was reduced from 600 to 400 mg; UPN 12 and 13, in whom DAS was switched to BOS; UPN 7, in whom DAS used as a first-line treatment was discontinued; and UPN 31, in whom PON used as a second-line treatment was discontinued. These results showed that TKI reduction, switching from the second-and third-generation TKIs to BOS or IM, and TKI discontinuation were associated with normalized TRPG, BNP, and blood pressure. Such positive findings occurred in 12 of 14 patients (86%). Conversely, TKI reduction was not associated with TRPG and BNP improvement in the remaining two patients (14%) (UPN: 40 and 41) who received NIL for >6 years.

Management of cardiotoxicity by discontinuation/switching of TKI in three representative cases

**Figure 4:**
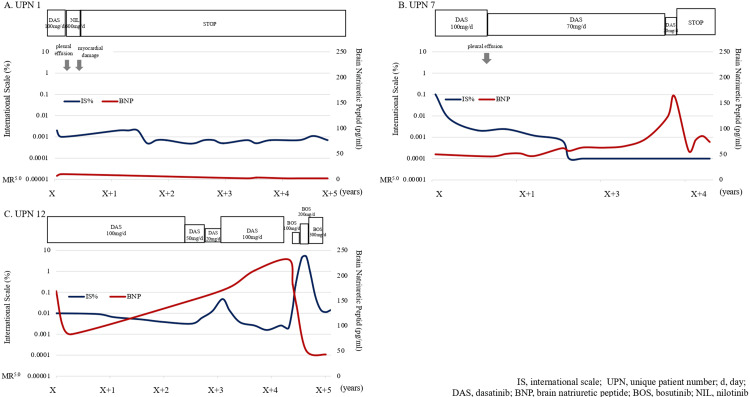
Control of cardiotoxicities via discontinuing/switching TKI in three representative cases The X-axis indicates time in years from the initiation of TKI therapy. A) UPN 1: Dasatinib was initiated as a second-line treatment but was switched to nilotinib because of pleural effusion. However, nilotinib was also discontinued because of myocardial damage. The patient achieved MR 4.5 at the time of nilotinib discontinuation. DMR has been maintained without TKI re-administration. B) UPN 7: MR 4.5 was achieved after the initial treatment with dasatinib. However, this drug was reduced and discontinued because of pleural effusion and elevated BNP. Subsequently, such symptoms improved. MR 4.5 has been maintained without TKI readministration. C) UPN 12: MR 4.5 was achieved after the initial treatment with dasatinib. However, this drug was switched to bosutinib because of elevated BNP. MR 4.0 was maintained, and BNP became normal. No other CVEs were observed.

The clinical course of these patients was also examined. In June 201X, four years after initiating DAS as a second-line treatment, UPN 1 (Figure 4A) experienced PE; thus, DAS was switched to NIL. In July 201X, the NIL was discontinued because of abnormal findings on UCG and T-wave inversion on ECG owing to myocardial damage. The patient achieved MR 4.5 at the time of discontinuation. DMR has been maintained for 75 months since the discontinuation; therefore, TKI re-administration is not required. PE and heart failure developed in UPN 7 during the treatment with second-generation TKIs; however, the patient's condition improved after TKI discontinuation (Figure 4B). The severity of cardiotoxicity improved after switching from the second-generation TKI to BOS in UPN 12 (Figure 4C). UPN 7 was diagnosed with CML in April 201X+1. DAS 100 mg/day was administered, and the patient achieved MR 4.5 in January 201X+3. However, PE and elevated BNP were observed; thus, it was reduced to 20 mg/day (Figure 4B). However, DAS was discontinued in July 201X+5 because BNP increased from 123 to 163 pg/mL. Subsequently, BNP decreased to 74.2 pg/mL. The patient maintained an MR4.5 without cardiotoxicity (Figure 4B). UPN 12 was diagnosed with CML in January 201X-2. DAS 100 mg/day was initiated (Figure 4C). The patient maintained an MR of 4.5 for >2 years, but BNP increased, and DAS decreased to 20 mg/day in April 201X+3. However, the patient failed to achieve an MR of 4.0 in January 201X+4. DAS was increased to 100 mg/day, achieving an MR of 4.5. However, BNP increased to 232 pg/mL, and DAS was switched to BOS 100 mg/day in April 201X+5. BOS was increased to 300 mg/day while monitoring the patient's tolerability. The patient maintained an MR of 4.0 without worsening CVEs (Figure 4C).

## Discussion

Treatment outcomes of CML-CP have dramatically improved after TKI development. The efficacies of the second- and third-generation TKIs for the treatment of this disease are higher than those of the first-generation TKIs [3,6,7]. Haguet et al. investigated patients with CML, and a meta-analysis was performed using the results of the early-phase TKI treatment. The results showed that the odds ratio for CVE onset is higher in the second- and third-generation TKI groups than in the IM group (i.e., 3.89, 2.68, and 1.61 in the NIL, DAS, and BOS groups, respectively) [18]. Cirmi et al. analyzed the adverse events during the TKI treatment in patients with CML using the FDA Adverse Event Reporting System. The results showed that the onset rates of CVEs in clinical practice were 59% (NIL), 21.2% (DAS), 14.4% (PON), 4.4% (IM), and 1.0% (BOS), indicating that it is higher in second-generation TKIs [19]. The onset rate of AOEs associated with IM is 0.6%, which is similar to that of age- and sex-matched general populations [11,18,19]. The onset rate of CVEs in the Bosutinib trial in First-line chronic myelogenous leukemia treatment (BFORE) trial, which compared BOS with IM, is similar between the two groups [3]. These results suggest that CVE incidence is lower in patients who receive IM or BOS than in those who receive second- and third-generation TKIs [20,21]. In clinical practice, patients with CML-CP are at high risk for lifestyle-related diseases and cardiovascular complications and have received TKIs for an extended period; moreover, their age is relatively high. Given such characteristics, treatment effects and adverse event profiles should be considered; thus, TKIs should be carefully selected. Furthermore, CVE monitoring via ECG, UCG, and BNP assessment is essential to continue the treatment of patients with CML-CP safely.

Results of clinical trials of TKIs for new-onset CML showed that the incidence of pericardial disease was 2.4% in BOS, 0% in IM (BELA trial [21,22]), 28% in DAS for pleural effusion (DASISION trial [1]), and 2% in PON for pericarditis (first-line ponatinib treatment for CML patients [9]). The ENESTnd trial [2], which compared NIL with IM, did not describe the incidence of pleural effusion with NIL treatment because the incidence of pleural effusion in patients enrolled in the trial was less than 4%. In this study, 16% (10/63) of patients developed PE, and monitoring of symptoms of PE by echocardiography and chest X-ray is necessary.

A study showed a significant prolongation of the QT interval immediately after IM in a first-generation TKI at pre- and post-TKI treatment [12]. The onset rate of QT interval prolongation is 25% in patients with IM-resistant CML-CP who receive NIL [12]. Care should be taken in prolonging the QT interval regardless of TKI generations. However, QTc of ≥500 ms was not observed in this study. The clinical trial results showed that the incidence of QTc >450 ms for each TKI was 6.1% for BOS, 7.7% or more for IM (BELA trial [22]), 2% for DAS (DASISION trial [1]), and 2% for PON (First-line ponatinib treatment for CML patients [9]). In the ENESTnd trial [2], there was no QTc >500 ms in CML patients treated with NIL, but QTc prolongation >60 ms from baseline was observed in 6.8% (300 mg bid) or 7.9% (400 mg bid) of NIL (ENESTnd trial [2]) compared with pretreatment. Although QTc >500 ms was not observed in this study, QTc was prolonged in 17 patients compared with pretreatment (median: 20 ms, range: 1-54 ms), and regular electrocardiographic monitoring may be necessary during TKI treatment. This observation may be partially explained by the fact that TKIs were reduced in patients who responded positively to treatment. In patients who experienced AP, cerebral infarction, and MI (UPN: 5, 39, 41, and 42), abnormal findings on ECG were seen 2-18 months before the onset of these diseases; therefore, ECG should be performed every three to six months [20] or when subjective symptoms occur.

In addition to diabetes, dyslipidemia, and smoking, hypertension is the main risk factor for coronary artery disease. Large meta-analyses have demonstrated that a 10 mmHg reduction in sBP reduces major cardiovascular events by approximately 20% and coronary heart disease by approximately 15-20% [23]. Blood pressure control is essential to prevent CVEs [23-25]. In this study, clinically significant cardiovascular events occurred in nine patients, with seven (details in Table 2) of them having hypertension. Of the seven patients, three (UPN: 30, 39, and 41) received antihypertensive drugs and blood pressure monitoring. Hence, TKIs were continued in these patients without dose modifications. A reduced TKI dose with antihypertensive drug treatment was continued in one patient (UPN: 26). The TKIs had to be changed in the other three patients because of clinically significant cardiovascular events (AP, UPN: 5 and 23; cerebral infarction, UPN: 31). It has been reported that PON is more likely to cause hypertension than IM or DAS because of its stronger [26]. According to the results of the clinical trial, hypertension occurred in 7.7% of BOS, 5.2% of IM (BELA trial [22]), and 30% of PON (First-line ponatinib treatment for CML patients [9]). NIL and DAS were not reported in the clinical trial results (DASISION trial [1] and ENESTnd trial [2]). Mulas et al. reported that the incidence of hypertension was 17% with ponatinib, 8% with nilotinib, 8% with dasatinib, and 5% with bosutinib [26]. Among patients who developed hypertension after starting TKIs, 68% (13/19) had a history of smoking, and 26% (5/19) had CKD, which may have contributed to hypertension in this study. It is also important to monitor blood pressure, give guidance on quitting smoking, and provide lifestyle guidance for CKD prevention. Blood pressure control is essential for patients who receive a TKI regardless of its type. In this study, the incidence of clinically significant cardiovascular events was higher in patients classified into the moderate- or severe-risk group based on the criteria of SCORE2 Risk Chart, SCORE2-OP Risk Chart, and Suita Score. CVE development was associated with moderate or higher scores on the SCORE2 Risk Chart or SCORE2-OP Risk Chart (Pearson's correlation coefficient φ=0.26, p=0.04) and tended to be associated with moderate or higher scores on the Suita score (φ=0.18, p=0.15). Regular blood pressure monitoring is essential as a preventive approach for lifestyle-related diseases. TKI can be continued successfully with aggressive co-morbidity management [27,28].

The CVE incidence rates are 2% and 5% in the IM and DAS groups, respectively (DASISION trial [1]); 3.6% and 16.5% in the IM 400 mg/day and NIL 300 mg BID groups, respectively (ENESTnd trial [2]); and 16% in the PON group (PACE trial [4]), thus showing a higher CVE incidence of the second- and third-generation TKIs [11]. Conversely, the incidence of IM-associated AOEs is 0.6%, which is similar to that of age- and sex-matched general populations [11,18,19]. The CVE incidence is similar between the BOS and IM in the BFORE trial [3] and tends to be lower in IM and BOS than in the newer-generation TKIs [11,18-21]. Results of a meta-analysis performed by Douxfils et al. showed that the onset rates of CVEs are 1.04% and 5.88% in the IM and second- and third-generation TKI groups, respectively, thereby indicating a significant high onset rate in the latter group [11]. In the ENESTnd trial, the CVE onset was associated with a NIL decrease in a dose-dependent manner [2]. Fujioka et al. showed that the incidence of VAEs in the Japanese population, particularly of IHD, is more frequently observed among patients treated with nilotinib than among those treated with imatinib or dasatinib [29]. The risk factors for PAH in patients who receive DAS are age and treatment duration [10,11]. The median age of patients in the subclinical cardiovascular abnormalities group was higher than that of those in the clinically significant cardiovascular events group at the initial examination. Such adverse events were improved after reducing the TKI dose; however, it was not associated with a decreased BNP in two patients (UPN: 40 and 41) who used the second-generation TKI for an extended period. Careful monitoring of CVE onset is required for older adults and patients receiving second-generation TKIs for an extended period (Figure 1; Tables 4-5). For patients with a history of CVE or who experience CVE during TKI treatment, the same dose reduction and discontinuation of TKI are recommended. Many of the patients in this study were treated with multiple TKIs, making it difficult to identify the TKI responsible for CVE. On the other hand, the incidence of CVE was reported to be 2% and 5% in IM and DAS in the DASISION [1] clinical trial for new-onset CML, 16.5% in NIL 300 mg BID in the ENESTnd trial [2], and 16% in the PACE trial [4] conducted after second-line CML therapy, indicating that CVE incidence varies among TKIs. Therefore, switching from second- and third-generation TKIs to IM or BOS may be a reasonable strategy in selected patients. In this cohort, the choice of TKI modification strategy (dose reduction, switching, or discontinuation) was individualized based on the type and severity of cardiovascular findings, the tolerability profile of each TKI, availability of alternative agents with favorable cardiovascular safety (IM or BOS), and the patient's molecular response status. TKI discontinuation was considered in patients who had achieved sustained deep molecular response, consistent with current treatment-free remission strategies [6,7].

In the STIM trial [6], TKIs were successfully discontinued in 40% of patients who maintained CMR for >2 years. In the De-escalation and Stopping Treatment of Imatinib, Nilotinib, or Sprycel in CML (DESTINY) trial by Clark et al. [30], TKIs were de-escalated to half the standard dose for 12 months before discontinuation; TFR was achieved in 72% of patients in the MR4 group and 36% in the MMR group. Selection of the first-line TKI, which is associated with the early achievement of DMR, is essential to achieve TFR. Furthermore, TKI switching and DMR duration are essential factors to achieve TFR in patients who fail to achieve DMR [11]. The use of second-generation TKIs is expected when aiming at achieving and maintaining DMR earlier and longer. To prevent treatment interruption because of CVEs, regular monitoring via cardiovascular examination (e.g., chest X-ray, ECG, UCG, and BNP evaluation) is essential. Patients with moderate or higher scores on the SCORE2 Risk Chart or SCORE2-OP Risk Chart tended to be correlated with hypertension (Pearson's correlation coefficient φ=0.25, p=0.05) and significantly correlated with elevated BNP (φ=0.27, p=0.03). Therefore, HT and BNP monitoring are considered to be useful.

Patients with CML in clinical practice have been treated with multiple TKIs and monitored for CVE with multiple tests. The existing CVE report is a study that reported results when monitoring for a single test result given a specific TKI. We retrospectively analyzed the results of multiple cardiovascular tests (ECG, UCG, TRPG, BNP, and cardiothoracic ratio monitoring) in CML patients treated with multiple TKIs. Our study revealed important findings to establish appropriate CVE management in CML patients treated with TKIs and strategies for TKI modification and dose adjustment in patients at high risk of CVE or with CVE.

Limitations of the study include the small number of patients and the retrospective single-center design, which may limit statistical power and generalizability. In addition, the minimum eligibility criterion of one month of follow-up may have limited the assessment of treatment response and delayed adverse events in some patients. Cardiovascular testing was performed as part of routine clinical practice rather than according to a predefined protocol, and biomarker and imaging assessments were not uniformly available for all patients, which may have introduced detection bias. In addition, the choice of TKI modification strategy was individualized according to the clinical context rather than determined by a predefined protocol, which may limit reproducibility across institutions. Multivariable adjustment was not performed because the limited number of severe cardiovascular events was insufficient to support stable multivariable modeling without a substantial risk of overfitting. In addition, no comparator group was included, and causal inference regarding the effect of TKI modification strategies on cardiovascular outcomes cannot be established. Furthermore, because many patients were exposed to multiple TKIs over the course of treatment, attribution of individual cardiovascular events to a specific TKI may have been difficult in some cases. The broad definition of cardiovascular findings, which includes subclinical abnormalities, may overestimate the overall incidence of cardiovascular events compared with studies using stricter endpoint definitions such as major adverse cardiovascular events (MACE). Prospective studies with standardized monitoring and larger cohorts are warranted.

## Conclusions

We retrospectively analyzed the frequency of CVE onset and the utility of cardiac function monitoring in 63 patients with CML-CP treated with TKIs. Regular monitoring, such as BNP assessment, UCG, and blood pressure measurement, was associated with early CVE detection, which may facilitate early intervention and safe continuation of CML treatment. These findings should be considered hypothesis-generating and reflect observational associations in a real-world cohort. In selected patients, TKI modification, including switching to BOS or IM, dose reduction, or discontinuation, was observed in association with improvement in cardiovascular parameters; however, causal inference cannot be made from this study. Multidisciplinary cardio-oncology collaboration may support long-term CML management through structured cardiovascular monitoring and timely referral to cardiologists for patients with emerging subclinical abnormalities or established cardiovascular risk factors.
